# Pain catastrophizing and its associated factors in parents of children after polydactyly or syndactyly surgery: a cross-sectional survey

**DOI:** 10.3389/fped.2026.1761055

**Published:** 2026-05-20

**Authors:** Weiyun Jiang, Yuehe Liu, Yue Chen, Ying Yang, Yiming Liu

**Affiliations:** 1 Department of Burn and Plastic Surgery, Children's Hospital of Nanjing Medical University, Nanjing, Jiangsu, China; 2 Department of Orthopedic, Children's Hospital of Nanjing Medical University, Nanjing, Jiangsu, China; 3 Department of Nursing, Children's Hospital of Nanjing Medical University, Nanjing, Jiangsu, China

**Keywords:** care, catastrophizing, children, nursing, pain, parents, polydactyly, syndactyly

## Abstract

**Background:**

Polydactyly and syndactyly are common congenital hand malformations in children, with surgical correction as the primary treatment. Postoperative pain in children can trigger pain catastrophizing in parents, which may impair family care quality and children's rehabilitation. This study aimed to evaluate the current status of pain catastrophizing and its associated factors in parents of children after polydactyly or syndactyly surgery.

**Method:**

A cross-sectional survey was conducted from February 2024 to October 2025, enrolling parents of children who underwent polydactyly or syndactyly correction surgery. Data were collected using the Chinese version of Parental Pain Catastrophizing Scale

**Results:**

A total of 206 parents were included. The total score of parental pain catastrophizing was 41.86 ± 5.19 points. The helplessness subscale score (range 0–24) was 18.74 ± 2.83, the rumination subscale score (range 0–16) was 13.25 ± 2.18, and the magnification subscale score (range 0–12) was 9.87 ± 1.65. Multiple linear regression analysis showed that child's age (*β* = –0.953), number of corrective surgeries (*β* = 2.517), parental gender (*β* = 3.342), place of residence (*β* = 2.965), marital status (*β* = 3.896), and pain knowledge training (*β* = 2.753) were independently associated factors (all *P* < 0.001), collectively explaining 42.3% of the variance (adjusted *R*² = 0.423, *F* = 22.157, *P* < 0.001).

**Conclusion:**

Parents of children after polydactyly or syndactyly correction surgery showed a relatively high level of pain catastrophizing. Child's age (negatively associated), number of surgeries, parental gender, residence, marital status, and pain knowledge training were identified as associated factors, with marital status showing the strongest association. Cross-dimensional comparisons of subscale scores are not interpretable due to different numbers of items. These findings identify key factors associated with parental pain catastrophizing, which may inform the development of multidimensional and individualized support strategies. Further longitudinal studies are needed to establish causality and explore the association of additional clinical variables.

## Introduction

Polydactyly and syndactyly are among the most common congenital hand malformations in children. Surgical correction remains the primary treatment to improve morphological and functional outcomes (1–3). However, the complexity of such procedures varies substantially: simple polydactyly resection causes minimal trauma, whereas complex corrections involving bone reconstruction and neurotendinous repair result in more severe and prolonged postoperative pain (4–6). Young children, especially those under 3 years of age, are unable to verbalize their discomfort, which not only complicates clinical pain assessment but also easily provokes emotional distress in caregivers (7–9).

Pain catastrophizing refers to a set of negative cognitive and emotional responses to pain, including rumination, magnification, and helplessness, and is particularly prominent among caregivers of children in the postoperative period (10, 11). Parents with high levels of pain catastrophizing may become overly sensitive to their children's pain, refuse necessary analgesia, or limit rehabilitation (12), thereby forming a vicious “pain–anxiety–poor care” cycle that impairs the child's overall recovery (13, 14). While pain catastrophizing has been examined in parents of children undergoing major surgeries such as cardiac or orthopedic procedures, the population with polydactyly or syndactyly is distinct due to generally shorter hospital stays, variable surgical complexity, and unique psychosocial stressors related to appearance and function. The specific status of pain catastrophizing in these parents has not been systematically clarified, nor have the key associated factors—including children's age, number of surgeries, and parents' sociodemographic characteristics—been fully identified. This leaves clinical practice with a lack of targeted family support and intervention strategies.

Therefore, this study aimed to clarify the level of pain catastrophizing in parents of children after polydactyly or syndactyly surgery, systematically analyze the associations of child- and parent-related characteristics with pain catastrophizing, and provide a scientific basis for individualized family care and optimized postoperative pain management. Given the exploratory nature of the study, no *a priori* hypotheses were formulated.

## Method

### Ethical approval

This study has been reviewed and approved by the Medical Ethics Committee of Children's Hospital of Nanjing Medical University (Ethics Approval No.: 202512028-1), and all research procedures strictly adhere to the Declaration of Helsinki (15). Prior to enrollment, investigators provided a detailed explanation of the study purpose, procedures, confidentiality principles, and voluntary participation rights. Participants were informed that they could withdraw at any time without affecting their child's care. After full understanding, written informed consent was obtained. All personal information and data were anonymized and used solely for academic research.

### Study design

A cross-sectional survey was adopted. Using convenience sampling, parents of children who had undergone surgery for polydactyly or syndactyly and met the inclusion criteria were recruited from our hospital from February 2024 to October 2025.

### Sample size calculation

Referring to the principles of sample size estimation for multiple linear regression analysis (16), the minimum sample size requirement was set as “number of independent variables × 15”. Combined with the core associated factors involved in the study, the initial estimated minimum sample size was 165 cases. Considering the questionnaire recovery rate and the elimination rate of invalid questionnaires (expected to be 15%), the sample size should be at least 190 cases to meet the power requirements of multiple linear regression analysis and various statistical tests.

### Study population

The inclusion criteria for this study were as follows: the child was diagnosed with polydactyly or syndactyly and had completed corrective surgery (assessed within 1–4 weeks postoperatively); the questionnaire filler was the biological parent or legal guardian of the child and the primary caregiver after surgery; aged ≥18 years old, with basic reading and comprehension abilities to complete the questionnaire independently; and voluntary participation in the study with signed informed consent. In our clinical setting, most participants completed the survey during the routine postoperative follow-up visit at approximately 2 weeks after surgery (82.5%), while the remainder completed it during rehabilitation sessions for complex cases involving tendon or bone reconstruction.

The exclusion criteria included: the child had other congenital malformations, chronic pain diseases, or severe organ dysfunction; the parent had a history of mental illness, cognitive impairment, or language communication disorder; the family had experienced major changes (such as the recent death of a relative, economic crisis, etc.) that might interfere with the assessment of psychological status; and those who refused to participate or could not cooperate in completing the questionnaire survey.

### Survey tools

A general information questionnaire was self-designed, covering: (1) child's gender, age, BMI, and number of corrective surgeries; (2) parent's gender, age, place of residence, educational level, marital status, monthly family income, and whether they had received pain-related knowledge training (single item: “Have you ever received any formal training or education about postoperative pain management in children?” with “Yes”/“No”).

The validated Chinese version of the Parental Pain Catastrophizing Scale (Parent PCS) (17) was used to assess the pain catastrophizing level of the children's parents, which is suitable for evaluating the cognitive and emotional responses of caregivers to pain after pediatric surgery. The scale consists of 13 items, including three dimensions: Rumination (4 items), Magnification (3 items), and Helplessness (6 items), using a 5-point Likert scale (0 = not at all, 1 = slightly, 2 = moderately, 3 = severely, 4 = extremely). The total score ranges from 0 to 52, with a higher score indicating a higher level of parental pain catastrophizing (18). In this study, the scale demonstrated good internal consistency, with a Cronbach's *α* coefficient of 0.912 for the total scale and dimension-specific *α* coefficients ranging from 0.832 to 0.889.

## Survey process and data collection

All investigators received unified training before the survey, covering research background, questionnaire instructions, communication skills, and ethical considerations. The survey was conducted by on-site distribution and immediate collection during postoperative follow-up visits or rehabilitation sessions. Investigators explained the study to eligible parents in a quiet environment, distributed questionnaires after informed consent, and provided standardized clarifications when needed. Completed questionnaires were checked on the spot for completeness; missing or erroneous items were corrected immediately. Valid questionnaires were coded and double-entered into EpiData 3.1 (EpiData Association, Odense, Denmark). Logical verification and data cleaning were performed to remove outliers and duplicates.

## Statistical analysis

IBM SPSS Statistics for Windows, Version 26.0 (IBM Corp., Armonk, NY, USA) was used. Continuous data are expressed as mean ± SD and categorical data as frequency (%). For bivariate analyses, independent-samples *t*-tests (binary variables) and one-way ANOVA (multilevel categorical variables) were used to compare pain catastrophizing scores across subgroups. Pearson correlation analysis was employed for continuous variables. Variables with *P* < 0.10 in bivariate analyses were entered into a multiple linear regression model (stepwise method, *α*-in = 0.05, *α*-out = 0.10) to identify independent associated factors. Child's age was entered as a continuous variable. Model assumptions were checked with collinearity diagnostics (VIF), Shapiro–Wilk test, Durbin–Watson test, and White test.

## Results

### Participant characteristics

A total of 206 parents were included. Their mean age was 33.52 ± 5.18 years (range 24–51); 138 (67.0%) were mothers. A total of 128 parents (62.1%) resided in urban areas, and 168 (81.6%) were married. Most participants (82.5%) completed the survey during the routine postoperative follow-up visit at approximately 2 weeks after surgery; the remainder completed it during rehabilitation sessions for complex cases involving tendon or bone reconstruction. Further details are presented in Table 1. Bivariate analyses showed that child's age, number of corrective surgeries, parental gender, place of residence, marital status, monthly family income, and pain knowledge training were associated with catastrophizing scores (all *P* < 0.05). Child's gender, BMI, parental age, and educational level were not significantly associated (*P* > 0.05). *post hoc* pairwise comparisons (Tukey's HSD) for significant categorical variables are presented in the footnotes of Table 1.

**Table 1:** Baseline characteristics of participants and unadjusted associations with parental pain catastrophizing total score in parents of children after polydactyly/syndactyly surgery (*N* = 206).

Characteristic category	Group	N (case number)	Constituent ratio (%)	Total score of pain catastrophizing (x¯ ± s)	Test statistic (t/F) with df	p	Post-hoc pairwise comparisons (Tukey's HSD)
Child's gender	Male	110	53.4	41.52 ± 5.31	t(204) = 0.582	0.561	–
	Female	96	46.6	42.29 ± 5.03			
Child's BMI (kg/m^2^)	<16	28	13.6	42.31 ± 5.24	F(3,202) = 0.935	0.425	–
	16–18.4	135	65.5	41.76 ± 5.18			
	18.5–21.9	36	17.5	41.98 ± 5.32			
	≥22	7	3.4	42.65 ± 4.97			
Parental gender	Male	66	32.0	38.74 ± 5.26	t(204) = 6.893	<0.001	–
	Female	140	68.0	43.56 ± 4.98			
Place of residence	Urban	121	58.7	40.01 ± 5.22	t(204) = 5.342	<0.001	–
	Rural	85	41.3	44.12 ± 5.03			
Educational level	Junior high school and below	68	33.0	42.89 ± 5.16	F(2,203) = 2.018	0.135	–
	Senior high school/technical secondary school	75	36.4	41.98 ± 5.23			
	College and above	63	30.6	40.73 ± 5.42			
Marital status	Married	170	82.5	40.98 ± 5.21	F(3,202) = 12.674	<0.001	Divorced vs. Married, *p* < 0.001; Widowed vs. Married, *p* < 0.001; Unmarried vs. Married, *p* = 0.184
	Unmarried	8	3.9	43.56 ± 4.98			
	Divorced	18	8.7	47.65 ± 4.32			
	Widowed	10	4.9	48.12 ± 4.15			
Monthly family income (CNY)	<3,000	45	21.8	43.78 ± 4.97	F(4,201) = 2.893	0.023	<3,000 vs. ≥15,000, *p* = 0.012; 3,000–5,999 vs. ≥15,000, *p* = 0.038
	3,000–5,999	68	33.0	42.56 ± 5.12			
	6,000–9,999	53	25.7	41.23 ± 5.34			
	10,000–14,999	25	12.1	40.56 ± 5.46			
	≥15,000	15	7.3	39.87 ± 5.51			
Pain knowledge training	Yes	76	36.9	40.25 ± 5.34	t(204) = 4.987	<0.001	–
	No	130	63.1	44.67 ± 4.78			

PCS, pain catastrophizing scale. Continuous variables (child's age, BMI, number of surgeries, parent's age) were analyzed with Pearson correlations (see Table 3).

### Pain catastrophizing scores

As shown in Table 2, the total pain catastrophizing score was 41.86 ± 5.19. The helplessness subscale score (range 0–24) was 18.74 ± 2.83, rumination (range 0–16) was 13.25 ± 2.18, and magnification (range 0–12) was 9.87 ± 1.65.

**Table 2:** Dimension scores of the parental pain catastrophizing scale in parents of children after polydactyly/syndactyly surgery (*N* = 206).

Dimension	Number of items	Theoretical range (min–max)	Mean subscale score (summed, x¯ ± s)	Observed range
Rumination	4	0–16	13.25 ± 2.18	4–16
Magnification	3	0–12	9.87 ± 1.65	3–12
Helplessness	6	0–24	18.74 ± 2.83	6–24
Total score	13	0–52	41.86 ± 5.19	13–52

The theoretical range refers to the minimum and maximum possible summed scores for each dimension (e.g., Rumination: 0–16). The mean subscale score is the summed score, not an item average. Direct comparisons across dimensions are not meaningful due to different numbers of items.

### Bivariate analyses

Pearson correlation analysis (Table 3) revealed that child's age was negatively correlated with the total pain catastrophizing score (r = –0.428, *P* < 0.001), while number of corrective surgeries was positively correlated (r = 0.385, *P* < 0.001). Parental age and child's BMI showed no significant correlations (*P* > 0.05).

**Table 3:** Pearson correlations between continuous variables and parental pain catastrophizing total score in parents of children after polydactyly/syndactyly surgery (*N* = 206).

Associated factor	Pain catastrophizing total score (r)	p
Child's age (years)	−0.428	<0.001
Child's BMI (kg/m²)	0.087	0.215
Number of correction surgeries	0.385	<0.001
Parent's age (years)	−0.095	0.178

### Multiple linear regression analysis

Variables with *P* < 0.10 in bivariate analyses were entered into the regression model. Six variables were retained in the final model (Table 4): child's age (continuous), number of corrective surgeries, parental gender, place of residence, marital status, and pain knowledge training. The model explained 42.3% of the variance (adjusted R² = 0.423, F = 22.157, *P* < 0.001). Collinearity diagnostics showed no serious multicollinearity (all VIF < 1.2). Marital status demonstrated the strongest association (*β* = 3.896), followed by parental gender (*β* = 3.342) and number of surgeries (*β* = 2.517); child's age was negatively associated (*β* = –0.953). Parents who were mothers, lived in rural areas, were divorced or widowed, had younger children, had children with more surgeries, or lacked pain knowledge training reported significantly higher catastrophizing levels (all *P* < 0.001).

**Table 4:** Multiple linear regression analysis of factors independently associated with parental pain catastrophizing in parents of children after polydactyly/syndactyly surgery (*N* = 206).

Independent variable	Assignment method	*β* coefficient	Standard error (SE)	t value	p	VIF
Constant term	-	39.258	2.286	17.173	<0.001	-
Child's age (years)	Continuous variable	−0.953	0.162	−5.883	<0.001	1.079
Number of correction surgeries	1 = 1 time, 2 = 2 times, 3 = ≥3 times	2.517	0.448	5.618	<0.001	1.052
Parent's gender	0 = Father, 1 = Mother	3.342	0.705	4.740	<0.001	1.118
Place of residence	0 = Urban, 1 = Rural	2.965	0.687	4.316	<0.001	1.093
Marital status	0 = Married/Unmarried, 1 = Divorced/Widowed	3.896	0.812	4.798	<0.001	1.146
Pain-related knowledge training	0 = Yes, 1 = No	2.753	0.647	4.255	<0.001	1.075

Model fitting indices: R^2^ = 0.443, Adjusted R^2^ = 0.423, F = 22.157, *P* < 0.001.

## Discussion

The parents in this study exhibited a relatively high level of pain catastrophizing (41.86 ± 5.19), comparable to or slightly higher than levels reported in parents of children undergoing major orthopedic procedures (19), though lower than those observed in parents of children with chronic pain (20). The pronounced helplessness subscale score may reflect gaps in pain management knowledge and limited coping resources. This pattern aligns with previous research on caregivers of children with congenital malformations, who are predisposed to catastrophizing cognitions due to persistent concerns about appearance, function, and long-term outcomes (21, 22).

Marital status was the strongest associated factor, with divorced or widowed parents showing substantially higher catastrophizing, underscoring the critical buffering role of spousal support. Female caregivers reported higher catastrophizing than males, consistent with prior findings linking heightened empathy and greater caregiving burden to increased emotional distress (23). Rural residence was associated with higher catastrophizing scores, potentially due to limited access to medical resources, less exposure to pain management education, and weaker family support systems (24). Parents who had received pain knowledge training demonstrated lower catastrophizing, highlighting the potential of educational interventions to modify maladaptive cognitions (25).

Younger child age and a higher number of surgeries were also independently associated with greater catastrophizing. Young children's limited verbal ability forces parents to rely on ambiguous behavioral cues, which may lead to overestimation of pain severity (26) and increased caregiving burden (27). Repeated surgeries may foster cumulative stress and learned helplessness (28). These findings point to the need for targeted attention toward parents of young children and those whose children undergo multiple procedures.

Clinically, these results suggest that support strategies could be tailored to the identified risk profiles. For instance, structured pain education using plain language and visual aids may especially benefit rural parents or those without prior training (29, 30). Skills-based strategies—such as comfort communication and distraction techniques—could enhance confidence in parents of young children or those facing repeated surgeries. Psychological support, including one-on-one counseling or family therapy, may be prioritized for female caregivers and those who are divorced or widowed (31, 32). However, these suggestions should be evaluated in future interventional studies.

Several limitations must be acknowledged. The cross-sectional design precludes causal inference. Time since surgery was not systematically recorded, and the timing of assessment (routine review or rehabilitation session) may have introduced variability. Baseline psychological status was not assessed, so it is unclear whether observed catastrophizing reflects pre-existing traits or postoperative responses. We did not collect data on children's postoperative pain severity, long-term pain behavior, surgical complexity, or recovery outcomes, all of which could influence parental catastrophizing. Convenience sampling from a single center also limits generalizability. Longitudinal, multicenter studies are needed to validate these findings, examine causal pathways, and explore the relationship between parental catastrophizing and children's recovery.

## Conclusion

This study showed that parents of children after polydactyly or syndactyly surgery experienced a relatively high level of pain catastrophizing. Marital status, parental gender, number of surgeries, child's age, place of residence, and pain knowledge training were identified as independent associated factors, with marital status exerting the strongest influence. These findings highlight the multidimensional nature of parental catastrophizing and suggest that support strategies should be individualized—addressing knowledge deficits, social support gaps, and caregiving burden. Given the cross-sectional design and the absence of data on children's pain severity and surgical complexity, causal conclusions cannot be drawn. Future prospective, multicenter studies are required to confirm these relationships and to evaluate the impact of tailored interventions on both parental distress and children's recovery outcomes.

## Data Availability

The original contributions presented in the study are included in the article/Supplementary Material, further inquiries can be directed to the corresponding authors.
